# Supplementary material for: Early turbulence and pulsatile flows enhance diodicity of Tesla’s macrofluidic valve
Source: Nat Commun. 2021 May 17;12:2884. doi: 10.1038/s41467-021-23009-y (PMC8128925; doi:10.1038/s41467-021-23009-y)
Supplement: Supplementary file 1 — Description of Additional Supplementary Files [file 41467_2021_23009_MOESM1_ESM.pdf]

REVIEWER COMMENTS

Reviewer #1 (Remarks to the Author):

This work studied a macrofluidic device using the historical design proposed by Nikola Tesla and demonstrated its application as a DC rectifier for fluctuating input flows. These results are novel, and the experimental approaches look solid and neat, which I believe will impact the general fluid mechanics society. I support its publication in Nature Communications with only minor comments to be addressed by the authors.

In the section "A quasi-steady model of the AC-to-DC converter," the authors predicted the effectiveness E_M of the rectifier by a resistor-network model, which was later compared to their experimental finding. However, the final formula for E_M [= U_{DC}/(4Af)] is identical to that used for computing the measured values, E, which can cause some confusion. I believe that the authors may want to use a different symbol for U_{DC} in the expression of E_M to differentiate it with the latter.

In the section "Unsteady forcing of a fluidic AC-to-DC converter," symbol "A" (for the AC amplitude) was used earlier for a different meaning (as the wetted area). Please consider using a different one.

Reviewer #2 (Remarks to the Author):

Authors investigate the transition flow in Tesla valves for constant and pulsating flow conditions. The paper is well written and easy to read. Below are the major technical concerns.

Authors indicate that the Tesla valve provides for 'hydraulic' resistance. It would be more appropriate to use general terminology, since the original Tesla valve was actually designed for gaseous flow. One suggestion is "fluid flow pressure losses". If authors are to investigate a macro-fluidic Tesla valve, it seems almost imperative for them to comment on the use of gases in this device. However, the manuscript does not take this to mind and thus does not do total justice to Tesla's work/vision.

The low transition Reynolds number can be attributed to the bifurcation and mixing effects occurring within the multi-staged Tesla valve. It seems that such effects would naturally spur turbulence at lower flow rates. In fact, the Reynolds number may be misused here, when treating the Tesla valve as a blackbox. This Reviewer believes that flow transition along the Tesla valve is a local phenomena that is not captured by using a "bulk" Reynolds number that uses entrance velocity. It would be worth inspecting local velocities at branch points in the structure to see if they are near the classical 2300 range. The title would lead one to believe that a new mechanism for turbulence has been discovered, when in reality, local values of Reynolds numbers corresponding to local turbulence is occurring.

The ability of Tesla valves to become more effective during oscillating flow was observed in the reference below - which the authors should consider:

Thompson, S.M., Ma, H.B., Wilson, C.A., 2011, “Investigation of a Flat-Plate Oscillating Heat Pipe with Tesla-Type Check Valves,” Experimental Thermal and Fluid Science, 35 (7), pp. 1265-1273. doi:

10.1016/j.expthermflusci.2011.04.014

There is some informal language which may hurt the paper's interpretability. For example, "fluidic traffic"...

Authors define a flow resistance term. It would be beneficial to see the term independent of channel cross-section area so it can be more easily used for comparing results with other studies.

The friction factor used in this study is claimed to hold accurate for laminar and turbulent flows. The surface roughness should have an effect on the turbulence, so how can authors neglect this effect?

Measurement uncertainty should be discussed and any error bars included in the plots.

One major issue with this study is the lack of discussion on how the number of valves will impact physics. A "true" Tesla valve design is used, but previous studies show that 1-2 Tesla valve stages are sufficient for microfluidics. Was Tesla's initial design with ~10 stages arbitrary? Hence, it is unclear how the measurements for this ~10 staged Tesla valve are relevant to application. More importantly, how do these results scale with stage number?

It is not clear how entrance and exit pressure loss effects were dealt with in this study. It seems having a 'straight-away' section prior to the Tesla valve would be needed to ensure a fully-developed boundary layer. How would the obtained results change with flow development at entry?

Authors state "The trend suggests yet higher diodicity for Re > 2000." However, this is speculative in nature and should not be included. It is possible that an asymptotic trend is realized at some point.

For Figure 3, why is the first Tesla valve stage "skipped" during reverse flow? It would be of value to know how the first valve is not operating for this flow regime selected.

The AC/DC analysis and analogy are a highlight of this paper. However, this Reviewer would like to see the effect of flow pulsation a single-unit Tesla valve. It is believed more physical meaning can be extracted for a single unit. The 'un-used' stages of the valve will dampen a lot of the pulsation. Also, is there a physically-relevant natural frequency worth commenting on?

Authors provide: "We speculate that this effect and the general mitigation of pulsatility as compared to quasi-steady
expectations are due to flow inertia, which tends to filter out fluctuations but which is absent from the quasi-steady framework." It would be better if authors can provide commentary that is confirmable by the experiments.

Authors state: "200 stated in Tesla’s patent seems well beyond reach." Have authors considered gaseous/compressible type flow? Again, it is believed that the initial Tesla patent discussed gaseous type flow.

Reference 10 needs revision so that Tesla is listed as last name.

Signed: Scott M. Thompson

Response to Reviewer #1

We thank the reviewer for this encouraging assessment and for the suggested clarifications. Below we address all suggestions with revisions to the manuscript.

> This work studied a macrofluidic device using the historical design proposed by Nikola Tesla and demonstrated its application as a DC rectifier for fluctuating input flows. These results are novel, and the experimental approaches look solid and neat, which I believe will impact the general fluid mechanics society. I support its publication in Nature Communications with only minor comments to be addressed by the authors.

We thank the reviewer again for this praise of the work, and we hope the changes detailed below shore the remaining issues.

> In the section "A quasi-steady model of the AC-to-DC converter," the authors predicted the effectiveness E_M of the rectifier by a resistor-network model, which was later compared to their experimental finding. However, the final formula for E_M [= U_{DC}/(4Af)] is identical to that used for computing the measured values, E, which can cause some confusion. I believe that the authors may want to use a different symbol for U_{DC} in the expression of E_M to differentiate it with the latter.

To correct this, we have modified the formula relevant to the model to eliminate the variables U_{DC}, A, and f. The revised text is colored red at the bottom of page 7.

> In the section "Unsteady forcing of a fluidic AC-to-DC converter," symbol "A" (for the AC amplitude) was used earlier for a different meaning (as the wetted area). Please consider using a different one.

To correct this, we have added a subscript “w” for variables representing quantities such as wetted volume, area and perimeter length. The revised text is colored red near the top of page 4.

Response to Reviewer #2

We thank the reviewer for this thorough and insightful assessment and for the many helpful recommendations throughout the report. Below we address all issues point by point and with corresponding revisions to the manuscript.

> Authors investigate the transition flow in Tesla valves for constant and pulsating flow conditions. The paper is well written and easy to read. Below are the major technical concerns.

We thank the reviewer for this encouraging assessment, and we hope the responses and revisions outlined below clarify the issues raised.

> Authors indicate that the Tesla valve provides for 'hydraulic' resistance. It would be more appropriate to use general terminology, since the original Tesla valve was actually designed for gaseous flow. One suggestion is "fluid flow pressure losses". If authors are to investigate a macro-fluidic Tesla valve, it seems almost imperative for them to comment on the use of gases in this device. However, the manuscript does not take this to mind and thus does not do total justice to Tesla's work/vision.

We thank the referee for raising this point, and we have now eliminated the word “hydraulic” except in the case of “hydraulic diameter”, a term commonly used regardless of the working fluid. Throughout the text, we now adopt more inclusive language such as “fluidic resistance”, “flow-induced pressure drop”, “resistance”, “friction”, etc. This is also in line with Tesla’s patent, which uses “fluid” or “medium” throughout the general descriptions of the device and “hydraulic”, “air” and “gas” in specific contexts.

Regarding the use of gases, we have now included this possibility in the discussion near the middle of page 9. The relevant text (colored red) indicates that gases may behave differently from liquids due to their compressibility but only at the extreme speeds needed to reach transonic and supersonic flow (order one or greater Mach number). It seems unlikely that such effects are important in most conceivable applications. For example, even the flow speeds in internal combustion engines are on the order of tens of meters per second, which is still quite low Mach number and hence flow-induced compressibility effects are weak. Thus, for the conditions expected in most applications, the fluidic properties of the conduit are expected to not depend explicitly on the fluid phase – as gases and (Newtonian) liquids alike obey the Navier-Stokes equation – but rather on flow regime, as characterized dimensionless quantities such as Reynolds number.

> The low transition Reynolds number can be attributed to the bifurcation and mixing effects occurring within the multi-staged Tesla valve. It seems that such effects would naturally spur turbulence at lower flow rates. In fact, the Reynolds number may be misused here, when treating the Tesla valve as a blackbox. This Reviewer believes that flow transition along the Tesla valve is a local phenomena that is not captured by using a "bulk" Reynolds number that uses entrance velocity. It would be worth inspecting local velocities at branch points in the structure to see if they are near the classical 2300 range. The title would lead one to believe that a new mechanism for turbulence has been discovered, when in reality, local values of Reynolds numbers corresponding to local turbulence is occurring.

We thank the reviewer for raising this potential interpretation for the early onset of turbulence. To assess this hypothesis, we closely examined the dye visualization Supplementary Video corresponding to reverse flow at Re $= 200$ and carried out point-wise measurements of the local flow speeds using a frame-by-frame analysis. We concentrated on the middle of channel, specifically in the vicinity of the 6th unit of the 11 in Tesla's conduit, where the unsteadiness leads to variations in the dye distribution and intensity that are advected and can be tracked. We manually measured speeds at 5 locations, as indicated on the attached image (flow from the right), gathering measurements at 10 different times for each location. (Note that the points A and A' are at analogous locations within the periodic array, and the measured speeds at these two sites were indistinguishable and thus lumped together.) The means and standard deviations, expressed relative to the bulk mean U, are as follows: $u_A/U = 1.3 \pm 0.2$, $u_B/U = 1.0 \pm 0.1$, $u_C/U = 1.1 \pm 0.2$ and $u_D/U = 1.4 \pm 0.1$. (The means tend to be greater than the bulk mean since most the chosen sites are in the middle of the respective lanes and away from walls, where the flows are slowed.) While the manual measurement is not as accurate as we would typically do with, say, PIV, the outcome is clear: The local speeds differ from one another and from the mean by less than 1.5 times (50%) and far less than the 10 times (1000%) needed to reach local Reynolds numbers in the thousands.

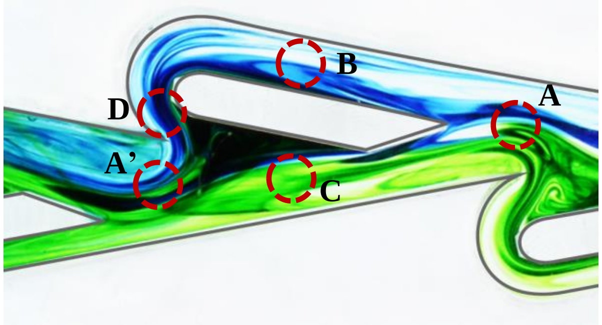

To clarify this point, we have added a new paragraph on page 5 (colored red), which is reproduced here for convenience: "In interpreting this early turbulence phenomenon, a concern may be that the Reynolds number defined here based on the mean speed inadequately captures the local flow conditions at various positions in the conduit. However, close inspection of the reverse flows in the Supplementary Video indicates that speeds at different sites along the central and diverted lanes are comparable to one another, with differences measuring less than 50%. Hence, the onset of turbulence at unusually low Re ≈ 200 cannot be attributed to local surges in flow speed significant enough to reach the conventional transitional value of Re ≈ 2000 for pipe flow. An alternative interpretation for the early onset of turbulence is given in our concluding discussions."

To the reviewer's larger point, we agree that the bifurcations and mixing induced by the structures inside the conduit must be responsible for the early onset. We would offer the following explanation. The main conduit and its internal structures are of the same scale, so in some sense the flow conditions are intermediate between internal and external flow. For external flows around bluff bodies, vortex shedding first occurs near Re ≈ 100, and the linear-to-quadratic transition in drag kicks in at the same point. (For example, a circular cylinder has laminar vortex shedding for Re $= 40$ to 150, and turbulence appears in the vortex wake for Re $= 150$ to 300.) The same sorts of phenomena likely occur locally in Tesla's channel around the intruding features. We have added a discussion paragraph spanning pages 9 and 10 covering these points: "Towards interpreting the apparently early onset of turbulence, we first note that the system might be viewed as having qualities of both internal and external flows. This duality stems from the fact that the conduit width

and its internal structures that 'invade' the flow are of the same scale. Said another way, the relative roughness is of order one. For external or open flow around a bluff body, intrinsic unsteadiness in the form of vortex shedding first appears at $\mathrm{Re} \approx 100$ and is accompanied by a transition from linear to quadratic scaling of drag with flow speed[6,44]. Analogous events would seem to occur for the local flows around the intruding structures in Tesla's device. Hence, the signatures of turbulence observed here may be viewed as arriving early from the standpoint of internal flows but as expected for external flows."

> The ability of Tesla valves to become more effective during oscillating flow was observed in the reference below - which the authors should consider: Thompson, S.M., Ma, H.B., Wilson, C.A., 2011, "Investigation of a Flat-Plate Oscillating Heat Pipe with Tesla-Type Check Valves," Experimental Thermal and Fluid Science, 35 (7), pp. 1265-1273. doi:10.1016/j.expthermflusci.2011.04.014

We thank the reviewer for bring this paper to our attention, which is now included as reference 65. We have cited the work on page 10 in the contexts of previous studies on unsteady flow conditions and heat transfer applications.

> There is some informal language which may hurt the paper's interpretability. For example, "fluidic traffic"...

This informal term has been corrected on page 1, and we have taken care to avoid potentially confusing language throughout the text.

> Authors define a flow resistance term. It would be beneficial to see the term independent of channel cross-section area so it can be more easily used for comparing results with other studies.

We thank the referee for raising this issue, and indeed the friction factor is the standard used for internal flows because it accounts for pressure drop across different geometries. A subtle but important point here is that the cross-section geometry (area or diameter D) comes in differently to fluidic resistance for laminar and turbulent flows. The Hagen-Poiseuille law indicates that the pressure drop scales as U/D^2 for laminar flow, but the pressure loss scales as U^2/D for turbulent flows. (These can be understood by balancing pressure gradients against viscous and inertial stresses, respectively, in the Navier-Stokes equation.) Conveniently, all of this accounted for by the friction factor, and these same dependencies show up as $f_D \sim 1/\mathrm{Re}$ for laminar flow and $f_D \sim \mathrm{Re}^0$ or constant for turbulent flow. If the referee has in mind some other measure of resistance that can account for geometry (as well as flow speed and fluid properties) across flow regimes, we would be happy to report on this quantity as well. For a more complete discussion of the friction factor, please see the comment below.

> The friction factor used in this study is claimed to hold accurate for laminar and turbulent flows. The surface roughness should have an effect on the turbulence, so how can authors neglect this effect?

The Darcy friction factor is a nondimensional form of resistance in which pressure drop is normalized based on geometrical and fluid scales relevant to turbulent flow. As such, and much like other force or pressure coefficients used in fluid dynamics, it is not accurate or inaccurate but

rather a recasting of the information that may be useful when comparing different systems. There is extensive literature showing that this quantity collapses data across slender conduits (channels, pipes and ducts) of different dimensions, across different (Newtonian) fluids and across flow speeds, and so this metric is very much the standard for internal flows. The formula for friction factor does not explicitly include aspects of roughness nor any details of shape beyond basic dimensions (L and D), but the measured value f_D at a given Re certainly reflects the roughness and all other geometrical aspects. The same is true of any force/pressure coefficient, *e.g.* drag coefficient depends on shape despite the formula not explicitly including geometric quantities beyond planform area. In fact, the main value of the friction factor and related coefficients is to remove expected dependencies on overall dimensions, fluid properties and flow speed so that what remains can be attributed to more nuanced aspects of shape. This is reflected in the plots of Fig. 2 showing that smooth pipes, rough pipes, and Tesla's conduit in each direction indeed yield different $f_D(\text{Re})$ curves. Hence, roughness and indeed all aspects of shape are not at all neglected but very much determine the $f_D(\text{Re})$ curve for a given conduit.

We are unsure if and how to incorporate this information in the text, but if the reviewer thinks aspects of this discussion would be worthwhile to include, we would follow any recommendations.

> Measurement uncertainty should be discussed and any error bars included in the plots.

We thank the referee for pointing out this oversight. Relevant to the steady forcing experiments, we now include discussions of the errors in the caption of Fig. 2 as well as in a new paragraph at the top of page 4 (red text). The relative errors are on the order of 1%, and the error bars are smaller than the symbol sizes for all data in Fig. 2a-d and so have been suppressed in these plots. For the unsteady or pulsatile forcing experiments, we conducted multiple measurements at each set of conditions, and all of these data are plotted together in Fig. 6. To give a better sense of the trial-to-trial variations, we have also included representative error bars (standard deviations) for several conditions in Fig. 6a and b. The relative errors tend to be on the order of 5%.

> One major issue with this study is the lack of discussion on how the number of valves will impact physics. A "true" Tesla valve design is used, but previous studies show that 1-2 Tesla valve stages are sufficient for microfluidics. Was Tesla's initial design with ~10 stages arbitrary? Hence, it is unclear how the measurements for this ~10 staged Tesla valve are relevant to application. More importantly, how do these results scale with stage number?

We thank the referee for raising the issue about the number of units, which is an interesting question that is addressed to a good degree in the literature. For example, the work of Mohammadzadeh et al. titled "Numerical investigation on the effect of the size and number of stages on the tesla microvalve efficiency" and cited as ref. 26 finds that Di increases with the number of units N (varied from 1 to 4) and the Reynolds number Re. Similar conclusions are drawn in "Numerical study of diodicity mechanism in different tesla-type microvalves" by Nobakht et al. (ref. 23), which shows diminishing growth of Di with increasing N. These same trends are seen in "Numerical investigation of multistaged tesla valves" by Thompson et al. (ref. 24), which explores 1 to 10 units and shows that Di displays asymptotic growth towards a plateau. These simulation studies use different Tesla-like geometries and so no quantitative correspondences are expected, but the trends are consistent. Experiments on this topic are missing

in the literature, so while there is certainly more work to be done, these previous studies seem to provide a clear picture for the dependence on N.

There are compelling reasons rooted in the flow physics for why we opted to focus on Tesla's original design of a long, slender conduit. Since our goals in this paper are more towards fluid dynamical mechanisms than applications, we wish to follow the well-established procedures and characterizations developed for pipes, ducts and channels and to thereby compare and contrast with regard to internal flow phenomena understood in these standard systems. A major consideration is that of entrance length, which is also the subject of the following comment and our response. It is well known that characterizations of flow resistance or friction can be contaminated by entrance effects associated with flow development, and there are consequently well-vetted standards for the length to diameter ratio (or slenderness) needed to ensure that such effects are negligibly small. As it happens, the recommendation relevant to turbulent flow is that $L/D > 40$, a criterion very nearly met by the value $L/D = 38$ for our realization of the valvular conduit. Hence, Tesla's design with its 11 units is special in the sense that results on this system can be understood as representative of all longer geometries. Shorter systems would become increasingly contaminated by entrance effects.

Ensuring that entrance effects are negligible is critically important in our interpretation that changes in the scaling of Hagen number or friction factor with Reynolds number can be linked to the laminar-to-turbulent flow transition. This association is only valid for sufficiently slender conduits, with shorter systems displaying the scalings associated with turbulence even for truly laminar flow. All of the above points are included in a new paragraph that appears near the middle of page 4 (colored red). In addition, we revised the introductory paragraph spanning pages 1 and 2 to alert the reader that our interest is in exploiting the procedures and analyses established for slender conduits.

The reviewer's question about the physics behind varying N or, equivalently, the conduit length, is an intriguing one. Our thought is that what is important is the length relative to an intrinsic fluid dynamical length scale. To better understand what this intrinsic scale might be, we carried out additional flow visualization experiments on Tesla's device at different Re. These experiments and observations are described in more detail in our responses below, and the new images are contained in the new Figure 4. What we see is that laminar flows penetrate the beginning of the conduit, but this penetration length depends on Re. For low Re before the laminar-to-turbulent transition, the laminar length permeates the whole conduit. For high Re after the transition, the laminar region is greatly reduced to a length measuring only 1 or 2 units, and turbulent and well-mixed flows dominate. At the transition, the laminar and turbulent segments are about equal as the flow transitions near the midpoint of the conduit. These observations, along with the association of turbulence with diodicity, suggest that conduits significantly shorter than Tesla's would require higher Re to reach similar values of Di. Longer conduits are expected to behave much like Tesla's, as turbulent flows would permeate the entire length. These inferences, while probably too speculative to be included in this work, are consistent with the results of "Numerical investigation of multistaged tesla valves" by Thompson et al. (ref. 24).

> It is not clear how entrance and exit pressure loss effects were dealt with in this study. It seems having a 'straight-away' section prior to the Tesla valve would be needed to ensure a fully-developed boundary layer. How would the obtained results change with flow development at entry?

We thank the referee for raising this question, and we hope our response to the comment above provides the answer. Namely, we have followed the standard procedures and criteria established for internal flows that ensure entrance effects are negligibly small by simply employing a sufficiently long, slender geometry. While we cannot definitively say how the addition of a straight-away section would affect the results, this would seem to lead to smaller diodicity for the composite system as compared to the pure valvular conduit. This is expected since the straight-away would add comparable resistance to flow in both directions, thus diluting the diodicity. We believe this effect may have contributed to some of the lower diodicity values reported in the literature, as discussed in the first complete paragraph on page 9.

> Authors state "The trend suggests yet higher diodicity for Re > 2000." However, this is speculative in nature and should not be included. It is possible that an asymptotic trend is realized at some point.

We agree, and the corrected text now reads (red text near bottom of page 4): "Future work should investigate the behavior for $\mathrm{Re} > 2000$."

> For Figure 3, why is the first Tesla valve stage "skipped" during reverse flow? It would be of value to know how the first valve is not operating for this flow regime selected.

We thank the reviewer for this insightful observation, which touches on the nature of the laminar to turbulent transition. In essence, the flow is transitional at the value $\mathrm{Re} = 200$, with flow in the first third of the conduit being laminar (giving the impression that the diverted channels are "skipped"), the middle portion is destabilized, and turbulent-like mixing occurs at the downstream end. To better illustrate this, we have carried out additional experiments visualizing the flows at Reynolds numbers just prior to and just after the transition. These images are included in the new Figure 4, where we have included an image of the transitional case of $\mathrm{Re} = 200$ to facilitate direct comparison. More specifically, the values of $\mathrm{Re} = 50$, 200 and 400 are selected to correspond to just before, in the middle, and just after the abrupt turn-on of diodicity shown in Fig. 2e. Below the transition, laminar and steady flow penetrates the entire channel. Above the transition, the flow is destabilized very near the beginning, and highly mixed flows occupy most of the conduit. The transitional case is intermediate, with laminar flow penetrating the upstream end of the conduit and well-mixed flows occupying the downstream end. These points are discussed in a new paragraph (red) near the middle of page 5.

> The AC/DC analysis and analogy are a highlight of this paper. However, this Reviewer would like to see the effect of flow pulsation a single-unit Tesla valve. It is believed more physical meaning can be extracted for a single unit. The 'un-used' stages of the valve will dampen a lot of the pulsation. Also, is there a physically-relevant natural frequency worth commenting on?

We thank the referee for this praise, and we agree that exploring a single-unit conduit would be interesting, informative and perhaps better aligned with applications. There are a few significant hurdles, both conceptual and practical. The physical interpretation of results on shorter conduits – especially those as short as one unit – would be clouded (and even dominated) by entrance effects, as discussed in more detail in the above responses. The entrance and exit portions of a single-unit device would be comparable to its valvular segment, and so the geometric details of these portions

may influence the results and dilute diodicity. Similarly, for an AC-to-DC rectifying circuit, we anticipate that the results will depend on the details of how the conduits are joined at nodal regions and how they connect to the AC forcing branch. In contrast, using Tesla's design ensures that the valvular segment dominates, both for the purely DC forcing experiments on an isolated channel and for the AC-to-DC rectifier consisting of 4 bridged channels. This allows for a clean comparison and analysis of the two systems, which we have done via a quasi-steady circuit model and which may be comprised for a single-unit device.

Towards better framing our emphasis on the conventional limit of long, slender conduits, we have revised the introductory paragraph spanning pages 1 and 2 (red text).

In addition, in pursuing a single-unit study, we have run into several practical hurdles associated with the covid pandemic. Most importantly, the university fabrication facilities (NYU Leslie eLab and Physics Experimental Shop) that we rely on for laser cutting and general machining have been closed for the fall 2020 semester. Unfortunately, we have just got news that there are also no plans to reopen for the spring semester. This has left us without a reliable means for manufacturing the new parts needed. Secondly, experimental research at NYU is currently subject to safety protocols that restrict researchers from working together in lab spaces. This presents a major challenge for the AC experiments, as these require attention to the piston/motor control, PIV imaging region, and camera control and data acquisition via a computer. These experiments were typically run with all 3 authors participating, and major changes would be needed to have all facets run by a single person.

For these reasons, we would respectfully ask that single-unit experiments be considered for a future study, which would also benefit from collaboration with experts in simulations. The present paper is unified in its focus on Tesla's original design, which has significant merits in that the results have clean physical interpretations in terms established phenomena for flow through long, slender conduits.

Regarding the apparently "unused" portion of the conduit, we hope this is now addressed by our new visualizations at different Re. Namely, laminar flows penetrate the upstream end, but for high Re the penetration length is quite short and unsteady flows permeate the bulk of the conduit. Hence, the majority of the conduit is operational with respect to valvular action.

Regarding a natural frequency or timescale in the system, this is exactly what is captured by the Womersley number $\mathrm{Wo}^2 \sim \rho f D^2/\mu$. Essentially, $\rho D^2/\mu = D^2/\nu$ is the timescale for momentum to diffuse across the width of the conduit, and this is being compared to the timescale (or frequency f) of the AC forcing. When Wo^2 is large as in our study, then the pulsatile forcing is so fast that the usual parabolic profile does not have time to develop, and the flow in the AC segment of the circuit is plug-like. These points are clarified with revisions (red) to the text near the top of page 7.

> Authors provide: "We speculate that this effect and the general mitigation of pulsatility as compared to quasi-steady expectations are due to flow inertia, which tends to filter out fluctuations but which is absent from the quasi-steady framework." It would be better if authors can provide commentary that is confirmable by the experiments.

To address this issue, we have modified the relevant text on page 8 to state that this hypothesis could be tested using models or simulations that include inertia.

> Authors state: "200 stated in Tesla's patent seems well beyond reach." Have authors

considered gaseous/compressible type flow? Again, it is believed that the initial Tesla patent discussed gaseous type flow.

We hope our earlier response and changes to the manuscript satisfactorily address this issue. To paraphrase, we have now included this possibility in the discussion near the middle of page 9, though most conceivable applications would not seem to involve the supersonic flow speeds needed to induce compressibility effects in gases.

Reference 10 needs revision so that Tesla is listed as last name.

Thank you for catching this error, which has been corrected.
