# Supplementary material for: Early turbulence and pulsatile flows enhance diodicity of Tesla’s macrofluidic valve
Source: Nat Commun. 2021 May 17;12:2884. doi: 10.1038/s41467-021-23009-y (PMC8128925; doi:10.1038/s41467-021-23009-y)
Supplement: Supplementary file 2 — Peer Review File [file 41467_2021_23009_MOESM2_ESM.pdf]

Description of Additional Supplementary Files

File name: Supplementary Movie 1

Description: This video visualizes the internal flow of water through Tesla's conduit in the forward and reverse directions at Reynolds number Re = 200. A fixed pressure difference is applied across the channel, and blue and green dyes are injected upstream of the conduit to reveal flow streaklines. In the forward direction, the flow is observed to be quite steady. Perturbing the inlet tubing helps to show the presence of flow, videos of which otherwise appear static. In the reverse direction, the flows are highly unsteady and lead to strong mixing.
